# Energy-Efficient Connected-Coverage Scheme in Wireless Sensor Networks

**DOI:** 10.3390/s20216127

**Published:** 2020-10-28

**Authors:** Yun Xu, Wanguo Jiao, Mengqiu Tian

**Affiliations:** College of Information Science and Technology, Nanjing Forestry University, Longpan Load 159, Nanjing 210037, China; yxu95@njfu.edu.cn (Y.X.); mqtian96@njfu.edu.cn (M.T.)

**Keywords:** wireless sensor networks, energy efficiency, sensor node scheduling, routing protocol

## Abstract

In the wireless sensor network, the lifetime of the network can be prolonged by improving the efficiency of limited energy. Existing works achieve better energy utilization, either through node scheduling or routing optimization. In this paper, an efficient solution combining node scheduling with routing protocol optimization is proposed in order to improve the network lifetime. Firstly, to avoid the redundant coverage, a node scheduling scheme that is based on a genetic algorithm is proposed to find the minimum number of sensor nodes to monitor all target points. Subsequently, the algorithm prolongs the lifetime of the network through choosing redundant sleep nodes to replace the dead node. Based on the obtained minimum coverage set, a new routing protocol, named Improved-Distributed Energy-Efficient Clustering (I-DEEC), is proposed. When considering the energy and the distance of the sensor node to the sink, a new policy choosing the cluster head is proposed. To make the energy load more balanced, uneven clusters are constructed. Meanwhile, the data communication way of sensor nodes around the sink is also optimized. The simulation results show that the proposed sensor node scheduling algorithm can reduce the number of redundant sensor nodes, while the I-DEEC routing protocol can improve the energy efficiency of data transmission. The lifetime of the network is greatly extended.

## 1. Introduction

The wireless sensor network (WSN) that is composed by many sensor nodes is applied to many fields, such as environmental monitoring, military reconnaissance, and so on [1,2]. These sensor nodes distribute in remote areas and they are responsible for sensing and data transmission, which consume lots of energy [3,4]. In practice, the battery capacity of the sensor node is limited and the realization of the energy replenishment is difficult [5,6]. To prolong the lifetime of the network, the limited energy of the sensor node should be utilized more efficiently.

In WSN, the lifetime of the network is often defined as the longest time that sensor nodes remain connected under the premise of ensuring network coverage. The target coverage is one of typical coverage categories, and multiple monitored target points (MTPs) coverage is an important realization way of the target coverage [7]. A large number of sensor nodes is used to detect these MTPs and transmit sensed data to the sink in order to obtain accurate and detailed data [8]. Generally, more sensor nodes sensing the same MTP means better coverage quality. However, using too many sensor nodes to monitor the same MTP will cause more redundant sensing data, which is wasteful and inefficient for the energy of the sensor node. If the number of the sensor node sensing the same MTP is reduced, then the corresponding sensing data are reduced. Subsequently, the energy that is consumed by sensing and transmission is also reduced. Additionally, these reduced sensor nodes can turn to sleep. When some sensor nodes are depleted, these sleeping nodes can be awakened to replace the exhausted sensor nodes and keep the coverage of the network. The network lifetime is prolonged. Hence, under the condition of the adequate network coverage, let all MTPs be covered by minimum number of sensor nodes, which is an efficient way of optimizing the network lifetime. Selecting the minimum number of sensor nodes to cover all MTPs has proved to be an NP-hard problem, which is also named as SCP (the set covering problem) [9]. In some works [10,11,12], the sensor node scheduling algorithms are proposed in order to reduce redundant sensor nodes, the energy wasted on the redundant coverage can be reduced significantly. However, these algorithms are highly complex and usually cannot obtain the optimal solution. That is, there is still small redundancy in the network. Furthermore, they did not consider the connectivity of the network, the nodes found in the minimum coverage set may not be able to communicate with each other. To address above problem, in the paper we proposed an algorithm based on the genetic algorithm to find the first minimum connected coverage set in an iterative manner. With the network running, the relevant sleeping nodes are constantly awakened according to the remaining energy and replace the dead nodes while ensuring the minimum number of working nodes. The lifetime of the network is effectively extended. The proposed algorithm can be easily extended to the rechargeable WSN.

The routing selection is also an important factor that influences the energy efficiency of the data transmission. A higher energy efficiency routing protocol can reduce energy consumption and improve the network lifetime [13]. Organizing sensor nodes into clusters can effectively reduce the number of data transmission and, thus, reduce energy consumption, especially in large scale WSN. Many energy-efficient routing protocols are designed on the basis of a cluster structure. In [14,15], the authors proposed efficient algorithms to establish the optimal routing protocol, and the energy consumed on routing can be reduced. However, the energy of the sensor node is not considered when selecting the cluster head. If the energy of the node is very low, being selected as the cluster head will accelerate its death. In addition, the distance between the node and base station is not considered. Based on the DEEC protocol, we jointly consider the remaining energy of the node and the distance between the sensor node and the base station to construct clusters with non-uniform size and avoid the use of low-energy nodes.

Although above works can enhance the lifetime of the network, they only optimize energy consumption from one aspect, node scheduling or routing optimization. In fact, the energy consumption is determined by the number of redundant sensor nodes and the choice of routing jointly. The scheme combining the sensor nodes scheduling with the routing protocol designing can further improve the energy efficiency and network lifetime. In this paper, we try to achieve higher energy efficiency of the network through jointing the sensor nodes scheduling with the routing protocol optimization. When comparing with previous works, both two practical factors are considered, which has not been proposed before. Firstly, an efficient heuristic solution that is based on genetic algorithm (GA) is proposed to solve SCP. Therefore, the sensing energy can be reduced. A scheme is also proposed to select sleep sensor nodes to replace dead nodes. The network lifetime can be improved. After determining the fewest sensor nodes that can cover all MTPs, in order to enable the large network to run reliably and efficiently, we then optimize the routing protocol. An improved routing protocol, named the Improved-Distributed Energy-Efficient Clustering (I-DEEC), is proposed. The probability of cluster heads (CHs) is adjusted, according to the distance to the sink, the residual energy, and the average energy of sensor nodes. The network is then divided into many uneven clusters in order to make the energy consumed on data communicating more balanced. Each CH node is responsible to send collected data from other sensor nodes to the sink using the I-DEEC. Meanwhile, the data communication way of sensor nodes around the sink is also optimized, so that more energy can be saved.

According to above discussion, our contributions are concluded as follows.

1. The scheme considering the scheduling of sensor nodes and optimization of the routing protocol jointly are proposed in order to improve the lifetime of the network.
2. Aiming at the multi-objective scheduling problem composed of three conflicting objectives: the largest network coverage, the least number of active nodes and awakening the sensor nodes with more residual energy, an Minimum Coverage Control Algorithm (MCCA) based on genetic algorithm is proposed to dynamically find the minimizing sensor set. Through activating the minimum number of sensor nodes to cover all MTPs, the problem of the redundant coverage can be avoided. At the same time, to keep the monitoring work, we also present the wake-up scheme to activate the sleep neighbor with high remaining energy to replace the exhausted node and keep the minimum active sensor node.
3. Considering the distance from sensor nodes to the sink and the energy distribution of the network, the I-DEEC routing protocol is designed to improve the clustering policy, optimize routing selection, and data transmission way. The sensor node can finds the most suitable way to send the data to the sink and the lifetime of the WSN is further prolonged.

The remaining part is organized, as follows. Section 2 shows some related works on the node scheduling and routing protocol optimization. Section 3 introduces the system model, including system network model, energy consumption model and problem formulation. To solve the set covering problem, Section 4 presents an minimum coverage Control algorithm based on genetic algorithm. The I-DEEC routing protocol is also proposed in Section 5 to make the communication more efficient. Section 6 shows the performance of proposed algorithms. At last, Section 7 concludes this paper.

## 2. Related Work

In this section, we introduce many related works regarding the efficient scheduling of sensor nodes and the optimization of the routing protocol.

### 2.1. Node Scheduling

In WSNs, scheduling can be used in the multiple access control layer and the network layer [16]. In this paper, we discuss the use of sensor node scheduling in the network layer. Scheduling sensor activities is an effective way to extend the life of wireless sensor networks. Only activating the necessary sensor nodes at a certain moment can save much energy. Various efforts have been made in order to optimize wake-up scheduling in sensor networks. These methods are generally divided into two categories. The first category is to find the largest number of disjoint coverage sets to extend the life of the network. The second type maximizes the total working time of disjoint cover sets to extend the life of the network [17]. The authors of [11] used GA to divide sensor nodes into some disjoint sets, each of them contains the minimum number of sensor nodes to cover all MTPs. Only one set keeps active at one time, and the remaining sets will enter sleep mode. Therefore, the lifetime of WSNs can be extended. However, the author assumed that the entire set will be discarded when there exist a sensor node in current active set runs out of energy. A new set will be activated in order to continue the sensing work. This will lead to the energy waste of sensor nodes, since other sensor nodes in the abandoned set may still have much residual energy. In [10], a sensor node scheduling method that is based on improved GA is proposed to achieve network coverage and connectivity. Sensor nodes are densely deployed in the network area, activating a group of sensor nodes with more residual energy for coverage. By introducing a new mutation operation, redundant sensor nodes can be dormant. Based on the ant colony algorithm, the authors of [12] proposed an algorithm to deal with the SCP. The energy of the network can be saved through activating sensor nodes in the minimum coverage set that satisfies the coverage requirement by turns. Then a local wake-up scheduling method is also proposed. Once an active sensor node runs out of energy, its neighbor nodes in the disjoint coverage set will be activated. The network performance can be indeed improved; however, the author did not consider network connection constraints and routing strategies.

### 2.2. Routing Protocol Optimization

The authors of [15] showed that cluster-based routing protocols can render the sensor network more energy efficient. The CH filters and aggregates the data sent by sensor nodes in same cluster, which can optimize the total amount of the sent data and reduce the distance of the communication. LEACH is a classic routing protocol based on the clustering structure [18,19]. Each cluster and CH are all elected dynamically in each round. However, the election of the CHs does not consider the remaining energy of the nodes. Sensor nodes with less residual energy may be selected, which will influence the performance of the network. Leach-centralized (Leach-c) is a centralized version of LEACH [20]. In Leach-c, each node sends its own location information and energy information to the BS, and the BS calculates the network average residual energy. During the cluster head election, only the node whose residual energy is greater than the network residual energy can become the cluster head. Therefore, in terms of packet transfer rate and energy consumption, Leach-c produces better results than LEACH. In [21], an improved LEACH protocol is proposed and clustering is carried out based on the principle of Voronoi graph. The sensor nodes in the same Voronoi graph become a cluster, and the ant colony algorithm is added to the protocol to optimize the multi-hop routing protocol. The proposed method does improve the energy efficiency of the node, but it also increases the complexity of the algorithm. Considering the remaining energy of the node, DEEC proposed in [22] modified the probability of CHs to avoid the early death of the sensor node. In [14], when it comes to the selection of the CH, to make the load of the network more even, the distribution of the energy is considered. At the same time, the optimal number of CHs in the WSN is demonstrated. The distance between sensor nodes and the sink is also an important factor influencing the network performance, which is, however, not considered. In this paper, we take the distance from sensor nodes to the sink and the energy distribution of the network into account. The I-DEEC routing protocol is also designed to further prolong the lifetime of the WSN.

## 3. System Model

In this section, we introduce the system model, including network model, energy consumption model, and problem formulation.

### 3.1. Network Model

Suppose that a monitoring area is a two-dimensional plane *A*, where A=m×n. There are *N* sensor nodes with different initial energy are randomly placed on the area *A*. The sensing radius and communication radius of each sensor node are $R_{s}$ and $R_{c}$, respectively. The coordinate of the sensor node $s_{j}$ is denoted by $(x_{j},y_{j}),1\leq j\leq N$, which is assumed to be known by the sink. The sensor node set is $S=\{s_{1},s_{2},\ldots ,s_{N}\}$. The set of MTPs is $P=\{p_{1},p_{2},\ldots ,p_{M}\}$, where $p_{k}$ is the *k*-th MTP located at $\left(x_{k}^{\prime },y_{k}^{\prime }\right),k\in \left[1,M\right]$, and *M* is the number of MTPs. These MTPs are distributed in the area *A*. In this paper, we assume that the sensor network is static. To guarantee that all MTPs be covered, *N* and *M* satisfy N≫M.

In order to ensure all the collected data be sent to the sink, the network must be fully connected. Besides, all the channels among all sensor nodes and the sink are reliable. That is, there is no transmission error and retransmissions. In [23], the author has proved the necessary condition for the network connectivity is that, if the communication radius $R_{c}$ of sensor nodes is not less than twice the perception radius $R_{s}$, the network can achieve full connectivity under the premise of full coverage. Let $R_{c}=2R_{s}$. Subsequently, as long as the network is fully covered, it can be assumed to be connected.

A binary variable $G_{j,k}$ is used to indicate whether the sensor node $s_{j}$ can cover the MTP $p_{k}$, which can be defined as

(1) $$G_{j,k}=\{\begin{array}{c} 1\;if\sqrt{{\left(x_{j}-x_{k}^{\prime }\right)}^{2}+{\left(y_{j}-y_{k}^{\prime }\right)}^{2}}\leq R_{s},\\ 0\;otherwise. \end{array}$$

In (1), $\sqrt{{\left(x_{j}-x_{k}^{\prime }\right)}^{2}+{\left(y_{j}-y_{k}^{\prime }\right)}^{2}}$ is the distance between the MTP $p_{k}$ and the sensor node $s_{j}$. If the distance is smaller than $R_{s}$, $G_{j,k}=1$ indicates that MTP $p_{k}$ can be covered by sensor node $s_{j}$. Because sensor nodes are distributed randomly, the MTP may be covered by *v* sensor nodes at the same time, this phenomenon can be expressed as

(2) $$G_{1,k}+G_{2,k}+\ldots +G_{v,k}=v.$$

It can be seen from (2) that, if MTP $p_{k}$ is covered, the following condition must be satisfied.

(3) $$\sum_{j=1}^{N}G_{j,k}\geq 1.$$

### 3.2. Energy Consumption Model

**Figure 1:**
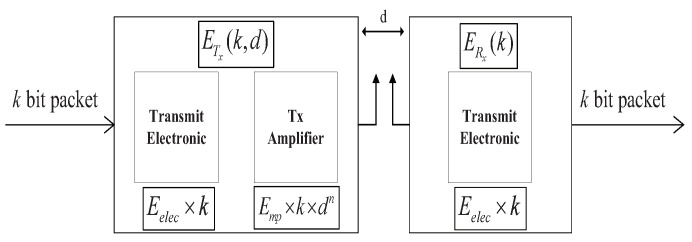
Energy consumption model.

According to [24,25], we assume that the energy of sensor nodes is consumed by sending, receiving, and fusing data. Figure 1 shows the relation among the energy consumption by each part.

The energy consumed by receiving *k* bits data is

(4) $$E_{R_{x}}\left(k\right)=k\times E_{elec},$$

where $E_{elec}$ represents the energy consumed on per bit of data by the transmitter or the receiver.

When a node transmits *k* bits data to a receiver with the distance *d*, the energy consumption can be written as

(5) $$\begin{array}{cc} E_{T_{x}}\left(k,d\right) & =E_{T_{x}-elec}\left(k\right)+E_{T_{x}-amp}\left(k,d\right)\\ & =\left\{\begin{array}{c} kE_{elec}+kE_{fs}d^{2},d<d_{0}\\ kE_{elec}+kE_{mp}d^{4},d\geq d_{0} \end{array}\right.\;, \end{array}$$

where $E_{T_{x}-elec}\left(k\right)$ is the consumed energy of transmitting data at the transmitter and $E_{T_{x}-amp}$ is the energy that is consumed by the transmitting amplifier [26]. According to the distance *d* between the sending node and the receiving node, the channel model can be divided into the free space model and multi-path fading model. $E_{f_{s}}$ and $E_{mp}$ are the energy loss coefficient of power amplifier in two models, respectively. $d_{0}$ is the distance threshold, $d_{0}=\sqrt{E_{fs}/E_{mp}}$. If the distance *d* is less than the threshold $d_{0}$, the free space model is used for power amplification loss. Otherwise, the multi-path fading model will be used.

For the CH, it is also responsible to fuse data comparing with other common nodes. The energy that is consumed on fusing *k* bits data by the CH can be expressed as

(6) $$E_{deal}=k\times E_{DA},$$

where $E_{DA}$ is the energy consumption of the CH fusing one bit data.

### 3.3. Problem Formulation

Monitoring the MTP consumes the energy of the sensor node. Because the battery capacity of the node is limited, once its energy is exhausted, the sensor node will die and the performance of the network will be influenced. Let τ be the lifetime of the WSN. Once there exist a MTP that cannot be covered by any sensor node, the network operation is assumed to be interrupted. The total working time of the network is the value of τ.

**Figure 2:**
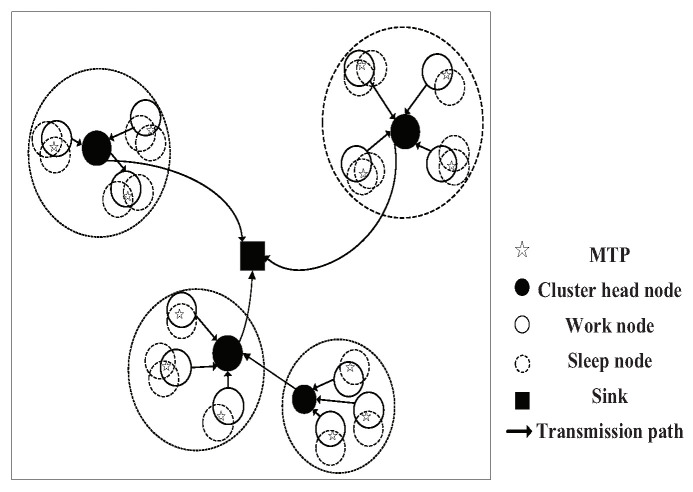
Network structure diagram.

When many sensor nodes are applied to detect one MTP, there will be redundant coverage, as shown in Figure 2. If these sensor nodes work at the same time, the network energy will be consumed too quickly. If one sensor node is used to monitor the MTP, when its energy is exhausted, the neighbor sleeping node can replace it. Such a cycle can extend the working time of the network. Therefore, as long as the minimum sensor nodes are found to cover all MTPs, the network lifetime can be maximized. Furthermore, it can be seen from (5) that the energy that is consumed by data transmission is related to the distance, an appropriate CH choosing policy can reduce the energy consumed on the long rang data transmission, and then the network lifetime can be further extended.

According to the above description, sensor nodes are distributed in a high density, which may lead to the redundant coverage. Thus, much energy will be wasted if all sensor nodes work simultaneously. Subsequently, the lifetime of the network cannot be optimal. At the same time, the collected data of sensor nodes are sent to the sink through single-hop or multi-hops, which also consumes the limited energy of sensor nodes. The energy efficiency of data transmission is closely related to the routing selection. Hence, the network life maximization problem can be divided into two sub-problems with regards to the sensor nodes scheduling and route protocol optimization.

First, the sensor nodes scheduling can be described as the set coverage problem (SCP), which is defined, as follows. Considering the limited energy of the sensor node, the redundant coverage should be avoided to prolong the lifetime of the network. The problem is how to find the minimum number of sensor nodes to cover all MTPs in the WSN, so that, the energy can be utilized more efficiently. Second, to optimize the route protocol and save more energy consumed on the communication, the energy saved maximization problem is proposed and described, as follows. Given a minimum coverage set, how to optimize the route protocol so that the network lifetime can be further extended. By considering these two sub-problems, the lifetime maximization problem can be described as

(7a)maxτ(7b)s.t.sj∈sminCover(7c)∑jGj,k×vj≥1,k∈[1,M](7d)e(sj)×vj>eth,vj∈{0,1}(7e)path(sj,sink)≥1

where $s_{minCover}$ is the set of sensor nodes, which is the solution of the optimization problem with the target of $\min\sum_{i=1}^{N}v_{i}$ under constraints (7c) and (7d). $v_{j}=1$ means that the sensor node $s_{j}$ is in the active state, while $v_{j}=0$ means that the sensor node $s_{j}$ is in the sleep state. Constraint (7b) indicates that the number of activated sensor nodes is minimized. (7c) ensures that each MTP $P_{k}$ can be covered by at least one node. The network works in rounds, (7d) shows that the residual energy of the activated node should be greater than the energy threshold $e_{th}$. Subsequently, the work time of each cycle can be longer. $p_{ath}\left(s_{j},sink\right)$ represents the path of the sensor node $s_{j}$ in $s_{minCover}$ to the sink, and (7e) indicates that all of the sensor nodes have at least one path to the sink. By optimizing routing, the energy saved of each path are maximized, so the lifetime of the network can be further prolonged.

## 4. Algorithm for the Set Coverage Problem

In this section, when considering the problem of the redundant coverage, through optimizing the sensor node scheduling, an efficient algorithm that is based on the genetic algorithm is proposed in order to solve the SCP and maximize the lifetime of the network.

### 4.1. An Overview of Genetic Algorithm

The GA is a classic global optimization search algorithm [27]. Here, we first briefly introduce the process of the GA. First, GA randomly generates a first generation population of several individuals (also known as chromosomes). Each chromosome represents a viable solution to the problem, usually represented by a string of numerical values, symbols, or alphabets. Second, the elite chromosomes that have higher fitness value are selected. The common selection operations include proportion fitness strategy, roulette strategy, and tournament strategy. Subsequently, these elite chromosomes will be crossed and mutated in a random way. Crossover is a random exchange of genes between two chromosomes to form two new chromosomes. There are various types of crossover, like one-point crossover, two-point crossover, and so on. The crossover rate $R_{c}$ is an important factor influencing the convergence speed of GA [28]. Mutation refers to changing some genes of chromosomes, it can improve the local search ability of the GA and maintain the diversity of population to prevent premature phenomenon.

### 4.2. Minimum Coverage Control Algorithm

Based on the genetic algorithm, we propose a Minimum Coverage Control Algorithm (MCCA) to solve the SCP. The proposed MCCA contains two parts: genetic operation and wake-up scheme. Through the iteration of the algorithm, the minimum coverage set $s_{minCover}$ can be found to monitor all MTPs. The set of other redundant sensor nodes is denoted by $s_{redCover}$. With the operation of the network, some sensor nodes in set $s_{minCover}$ will be out of energy. To keep full MTPs covered, the proposed wake-up scheme will wake up the optimal node in set $s_{redCover}$ to replace the dead node. Accordingly, the lifetime of the network is prolonged.

**Figure 3:**
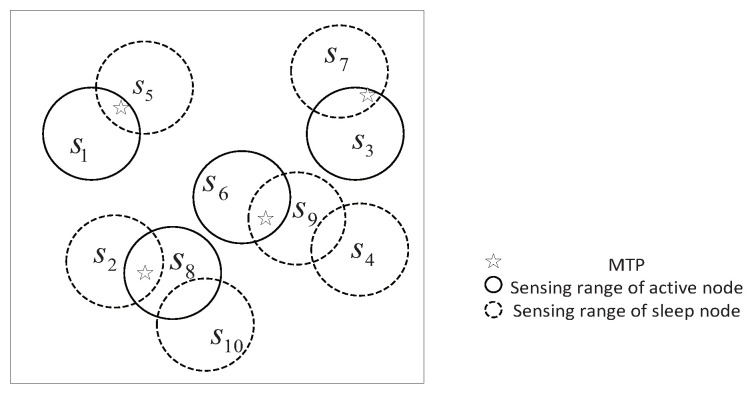
Coding example network.

The length of the chromosome is the same as the number of deployed sensor nodes in the network. The $k_{th}$ gene value $g_{i}$ signifies whether the sensor node $s_{i}$ selected as a active or sleeping node in current round. There are 10 sensor nodes $\left\{s_{1},s_{2},\ldots ,s_{10}\right\}$ randomly distributed in the sensing area to monitor four MTPs, as shown in Figure 3. Therefore, the length of the chromosomes for this network is ten and the string of the chromosome 1010010100 is used to represent the state of 10 sensor nodes. It can be observed that the gene value at position 6 is 1 (i.e., $g_{6}=1$), which implies that the sensor node $s_{6}$ is selected as active node in current round. Similarly, the sensor node $s_{1},s_{3}$ and $s_{8}$ are also the active node in current round. The gene value of $g_{2},g_{4},g_{5},g_{7},g_{9}$ and $g_{10}$ is 0, which implies that the sensor nodes $s_{2},s_{4},s_{5},s_{7},s_{9}$ and $s_{10}$ will be in sleep mode.

#### 4.2.1. Genetic Operation

The evolutionary process of the GA includes selection, crossover, mutation, and fitness function. The chromosomes are evaluated by the fitness function. Define the $\ell_{k,j}$ as the working state of node $s_{j}$ in the *k*th chromosome, which is assumed as 0 or 1. The coverage vector w(k) of the chromosome *k* can be expressed as the vector below,

(8) $$\left\{\begin{array}{c} {}[\left(\ell_{k,1}G_{1,1}\right)\cup \left(\ell_{k,2}G_{2,1}\right)\cup \cdots \cup \left(\ell_{k,N}G_{N,1}\right)],\\ \left[\left(\ell_{k,1}G_{1,2}\right)\cup \left(\ell_{k,2}G_{2,2}\right)\cup \cdots \cup \left(\ell_{k,N}G_{N,2}\right)\right],\\ \vdots \\ \left[\left(\ell_{k,1}G_{1,M}\right)\cup \left(\ell_{k,2}G_{2,M}\right)\cup \cdots \cup \left(\ell_{k,N}G_{N,M}\right)\right], \end{array}\right\}$$

In (8),$\left(\ell_{k,j}G_{j,m}\right)$ represents the coverage of the *j*th sensor node on the *k*th chromosome to the *m*th MTP. When $\left(\left(\ell_{k,1}G_{1,M}\right)\cup \left(\ell_{k,2}G_{2,M}\right)\cup \cdots \cup \left(\ell_{k,N}G_{N,M}\right)]=1\right)$, it means that the *m*th MTP is covered by at least one sensor node. In this paper, we assume that the channel is ideal. In the full connected network, the sensing data from the *m*th MTP can be successfully received by the sink. However, in practice, the wireless channel is unreliable, more than one sensor node with better channel quality is activated to cover one target. Subsequently, the SCP becomes a complicated cross-layer optimization problem, and this is out of the scope of this work.

The fitness function is proposed by considering the objectives, as follows.

Objective 1, the coverage rate of the MTP $\varepsilon^{k}$ should be largest. Subsequently, the performance of the network can be ensured.

(9) $$f_{1}=\max\varepsilon^{k}=\frac{{\left||w\left(k\right)|\right|}^{2}}{M},$$

where ||w(k)|| is the Euclidean norm, ${\left||w\left(k\right)|\right|}^{2}$ represents the number of MTPs that can be covered by the sensor nodes on the *k*th chromosome.

Objective 2, the number of active nodes on the *k*th chromosome, should be the least while the objective 1 is ensured. In other words, the sensor node dormancy rate η should be the largest value. Therefore, much more energy can be saved.

(10) $$f_{2}=\max\eta =1-\frac{\sum_{i=1}^{N}s_{i}}{N},v_{i}=1,\;$$

where $\sum_{i=1}^{N}s_{i}$ represents the number of active nodes.

Objective 3, the selected sensor nodes in set $s_{minCover}$ should have sufficient energy to serve up to a certain rounds.

(11) $$f_{3}=\max\frac{E_{r}}{E_{total}},$$

Let $E_{total}$ be the sum of the initial energy of all sensor nodes and $E_{r}$ be the maximum remaining energy of all sensor nodes.

We define f(k) as the fitness function of *k* chromosome. According to the weighted GA, the total objective function is defined as the weighted sum of mentioned objective functions. The fitness function should be maximized to achieve the better performance of the network, which is shown as

(12) $$\begin{array}{c} \max f\left(k\right)=\lambda_{1}\cdot \left(f_{1}\right)+\lambda_{2}\cdot \left(f_{2}\right)+\lambda_{3}\cdot \left(f_{3}\right)\\ =\lambda_{1}\cdot \left(\frac{{\left||w\left(k\right)|\right|}^{2}}{M}\right)+\lambda_{2}\cdot \left(1-\frac{\sum_{i=1}^{N}s_{i}}{N}\right)+\lambda_{3}\cdot \left(\frac{E_{r}}{E_{total}}\right) \end{array}$$

where $\lambda_{1}$, $\lambda_{2}$ and $\lambda_{3}$ are weighting coefficients and $\lambda_{1}+\lambda_{2}+\lambda_{3}=1$. It can be noticed that the larger the value of the objective function f(k), the better the goodness of the chromosome.

After achieving the fitness function, the process of determining the minimum coverage set is shown, as follows. Firstly, the tournament selection strategy is used to randomly select 10 chromosomes from the population (each chromosome has the same probability of being selected). The optimal chromosome with the maximum fitness will be put into the next generation population. Such an iteration will stop until the new population size is equal to the original population size. Secondly, randomly choose two parent chromosomes from the current population and use one-point crossover to cross them to produce new child chromosomes. Subsequently, unlike the conventional mutation stage that selects some gene randomly to change its value, we propose a novel mutation operation to accelerate the speed of the convergence. Through finding redundant or unnecessary active nodes and mutating its gene value from 1 to 0, the number of active sensor nodes can be considerably reduced.

#### 4.2.2. Local Wake-up Scheme

With the continuous operation of the WSN, some sensor node will run out of its energy. The wake-up strategy is also proposed to ensure the full coverage of MTPs. Through waking up the appropriate sensor node from set $s_{redCover}$ to replace the dead node, MTPs can be detected continuous.

The local wake-up scheme is divided into two parts. Firstly, define the set of all MTPs as $c_{1}\left(s_{i}\right)$ and the set of the remaining MTPs, except for that which cannot be monitored by nodes as $c_{2}\left(s_{i-d}\right)$, respectively. The MTP that cannot be monitored will be found by Boolean exclusive disjunction between sets $c_{1}\left(s_{i-d}\right)$ and $c_{2}\left(s_{i-d}\right)$, which is shown as

(13) $$c(s_{i}{)}_{uncovered}=c_{1}\left(s_{i-d}\right)⊕c_{2}\left(s_{i-d}\right).$$

Secondly, for the selected sensor node $s_{j}$ from set $s_{redCover}$, it should satisfies (i) the distance $d_{ij}$ between the selected sensor node $s_{j}$ and the dead node $s_{i}$ is no greater than the communication radius $R_{c}$, and (ii) the distance $d_{jm}$ between the selected sensor node $s_{j}$ and the corresponding *m*th MTP is no greater than the sensing radius $R_{s}$. These two constraints are shown, as follows

(14) $$\left\{\begin{array}{c} d_{ij}=\sqrt{{\left(x_{i}-x_{j}\right)}^{2}+{\left(y_{i}-y_{j}\right)}^{2}}<R_{c},\\ d_{jm}=\sqrt{{\left(x_{j}-x_{m}^{\prime }\right)}^{2}+{\left(y_{j}-y_{m}^{\prime }\right)}^{2}}<R_{s}. \end{array}\right.$$

Define the set of all sensor nodes in set $s_{redCover}$ that satisfy (13) as $s_{sect}$. Subsequently, select the optimal sensor node that has maximum residual energy comparing with others from set $s_{sect}$ to monitor the *m*th MTP.

The time complexity of the MCCA is bounded in O(N·M+K·ρ·N), where *N* is the number of sensor nodes, *M* is the number of the MTPs, ρ is the number of chromosomes, and *K* is the size of the population. First, the MCCA needs to calculate the distance between sensor nodes and MTPs. There are a total of NM operations, so the complexity of the first step is O(N·M). In genetic operations, we need to traverse *N* sensor nodes on each chromosome. Because there are a total of *K* populations and ρ chromosomes in each population, the complexity of the second step is O(K·ρ·N). These two operations run independently, thus the complexity of MCCA is bound in O(N·M+K·ρ·N). More details can be found in Algorithm 1.

**Algorithm 1** MCCA.
Input: The population is $Pop_{k}$, Let $chrom_{i}$ be the i−th individual in $Pop_{k}$, and *N* be the length of $chrom_{i}$. where $chrom_{i}$ is a binary string composed of $\ell_{i,j}$. The number of chromosomes is ρ.
Output: The best chromosome.
  1:Calculate the f(k);  2:Select elite individuals from initial population according to f(k);  3:One-point crossover;  4:Then use directed mutations to speed up convergence.  5:**for**i=1 to ρ **do**  6:  **for** j=1 to N **do**  7:   $f_{1}\left(i\right)=fit\left(chrom_{i}\right)$;  8:   In $chrom_{i}$, Let allele 1 mutate to 0.  9:   $f_{2}\left(i\right)=fit\left(chrom_{i}\right)$; 10:   **if** $f_{1}\left(i\right)1<f_{2}\left(i\right)$ **then** 11:    Let allele 1 mutate to 0. 12:   **end if** 13:  **end for** 14:
**end for**
 15:Whether the termination condition is met, output the best individual or return to step 1. 16:**if** node $s_{i-d}$ has exhausted its energy **then** 17:  wake up redundant from $s_{sect}$. 18:
**end if**



## 5. Algorithm for the Energy Saved Maximization Problem

After optimizing the sensor node scheduling, to further prolong the lifetime of the network, a new routing protocol that is based on DEEC is designed which can reduce the energy consumption and further improve the lifetime of the network.

### 5.1. DEEC Protocol Description

To best illustrate our proposed routing protocol, we provide a brief introduction of the DEEC protocol firstly. The DEEC is an efficient distributed clustering routing protocol [29]. The energy consumption of the CH is much higher than other normal nodes. To balance the energy load of nodes, in each round, the CH will be dynamically selected according to the ratio of the remaining energy to the average remaining energy of the network. In practice, there are many factors that influence the node energy consumption. That is, the energy consumption rates of different sensor nodes are different. Thus, the CH selection should consider not only remaining energy, but also the energy consumption rate. However, the DEEC only takes the remaining energy into consideration. We propose a new improved DEEC protocol by considering more factors that influence the lifetime of the sensor node.

### 5.2. Improved DEEC Protocol

By considering the energy of the sensor node and the distance to the sink comprehensively, we improve the DEEC from three aspects: the probability of the CH selection, the cluster size, and the communication way of sensor nodes around the sink.

#### 5.2.1. Adjust the Probability of CHs

For the DEEC protocol, sensor node $s_{i}$ generates a random number between [0,1] and compares it with the threshold T(n). If the random number is less than T(n), then this node will be selected as the CH [30]. The T(n) is represented as

(15) $$T\left(n\right)=\left\{\begin{array}{cc} \frac{p_{i}}{1-p_{i}\left(r\;\;\mathrm{mod}\;\;\frac{1}{p_{i}}\right)} & if\;n\in G\\ 0 & otherwise, \end{array}\right.$$

where $p\left(i\right)=\frac{E_{i}\left(r\right)}{\bar{E}\left(r\right)}$. $E_{i}\left(r\right)$ is the residual energy of the sensor node $s_{i}$ and $\bar{E}\left(r\right)$ is the average remaining energy of all the sensor nodes in the *r* round. *r* is the current number of rounds, *G* is the set of nodes that have not been selected as CHs. From (14), it can be found that only the residual energy of sensor nodes is considered.

The CH is responsible to fuse and transfer data that are sensed from other nodes; therefore, it should have enough residual energy. At the same time, because the distance between the CH and the sink is also an important factor influencing the energy consumption, to save more energy, the distance to the sink should be also considered. As described above, the probability of each CH $CH_{i}$ selection can be modified as

(16) $$T\left(CH_{i}\right)=T\left(n\right)*\left(\alpha *\frac{E_{i}\left(r\right)*E\left(i\right)}{\bar{E}\left(r\right)*E(a)}+\beta *D\right)$$

where E(i) is the initial energy of the node, E(a) is the average energy of all sensor nodes in the WSN. $D=(1-\frac{d\left(i,\sin k\right)-d_{\max}}{d_{\max}-d_{\min}})$. d(i,sink) represents the distance from node $s_{i}$ to sink, $d_{\max}$ and $d_{\min}$ are the maximum and minimum distance from node $s_{i}$ to sink, respectively. α and β are the weight coefficients and α+β=1. When α is a larger value, the residual energy of sensor nodes is a more important factor influencing the CH selection. When β is greater than α, the sensor node that nears the sink has more opportunity to be chosen as the CH.

#### 5.2.2. Uneven Clustering

To more reasonably divide the network, the uneven cluster structure is constructed. For the CH around the sink (which is defined as the CHA), except for processing the data from the cluster, it is also responsible to transmit the sending data from other CHs far away from the sink. Therefore, this kind of the CH has much higher energy consumption rate than others. To avoid the early death of the CHAs, their energy consumption load should be released, which means that the cluster size of the CHAs should be smaller. While, for the CH away from the sink, since the energy consumption rate of them is small, the cluster size of the CHs can be increased. As described above, the calculation of the cluster radius is defined as

(17) $$R_{c}=\left(1+\alpha \frac{E_{i}\left(r\right)}{\bar{E}\left(r\right)}-\beta \frac{d_{\max}-d\left(i,\sin k\right)}{d_{\max}-d_{\min}}\right)*R_{r}.$$

where $R_{r}$ is assumed as a constant, e.g, 40 m.

#### 5.2.3. The Communication Way of Sensor Nodes around the Sink

**Figure 4:**
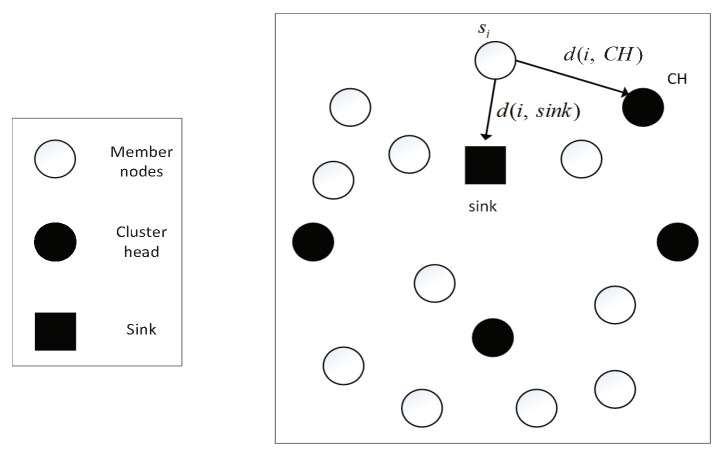
Cluster formation.

When CH in the network is selected and the CH broadcasts a message to other nodes, the non-cluster head node $s_{i}$ finds the nearest CH according to the received signal strength, as shown in Figure 4. If the sensor node $s_{i}$ joins the cluster with CH as the cluster head, the message will go through multi-hop or long-distance communication, which will consume a lot of energy. When comparing the distance d(i,sink) from the sensor node $s_{i}$ to the sink and the distance d(i,CH) from sensor node $s_{i}$ to its CH, if d(i,sink)<d(i,CH), sensor node $s_{i}$ will communicate with sink directly. Thus, the energy that is consumed of sensor nodes around the sink can be reduced.

The time complexity of the I-DEEC is bounded in $O(n^{2}+r\cdot n\cdot c)$, Where *r* is the number of rounds, *n* is the number of sensor nodes, and *c* is the number of cluster heads. First of all, we need to judge the distance between every two of the *n* sensor nodes, which is executed ((n−1)+(n−2)+⋯+2+1) times in total, so the complexity of the first step is $O(n^{2})$. The network runs a total of *r* rounds. We decide whether the sensor node joins the cluster group according to the distance between the sensor node and cluster head. There are a total of (r·n·c) operations, so the complexity of the second step is O(r·n·c). These two operations run independently, and their sum is I-DEEC complexity, so the complexity of I-DEEC is bounded in $O(n^{2}+r\cdot n\cdot c)$. Algorithm 2 outlines more details.

**Algorithm 2** I-DEEC.Input: C A set of sensor nodes in $s_{redCover}$ from Algorithm 1.Output: The path that consumes the least energy for data transmission.
  1:Calculate d(i,sink)  2:**for** For i=1 to *r* **do**  3: $p_{i}=\frac{E_{i}\left(r\right)}{\bar{E}\left(t\right)}$
  4: $T\left(CH_{i}\right)=T\left(n\right)*\left(\alpha *\frac{E_{i}\left(r\right)*E\left(i\right)}{\bar{E}\left(r\right)*E_{a}}+\beta *D\right)$
  5: where $D=(1-\frac{d\left(i,\sin k\right)-d_{\max}}{d_{\max}-d_{\min}})$.  6: *t*=Random number  7: **if** $T(CH_{i})<t$ **then**  8:  $CH\leftarrow n_{i}$
  9:  $R_{c}=\left(1+\alpha *\frac{E_{i}\left(r\right)}{\bar{E}\left(r\right)}-\beta *D\right)*R_{r}$
 10:  Calculate d(i,CH) 11: **end if** 12: **if** d(i,CH)<d(i,sink) **then** 13:  Select CH and join the cluster 14: **else** 15:  Nodes do not participate in the cluster 16: **end if** 17:
**end for**



### 5.3. Flow Chart of the Combination of Two Algorithms

**Figure 5:**
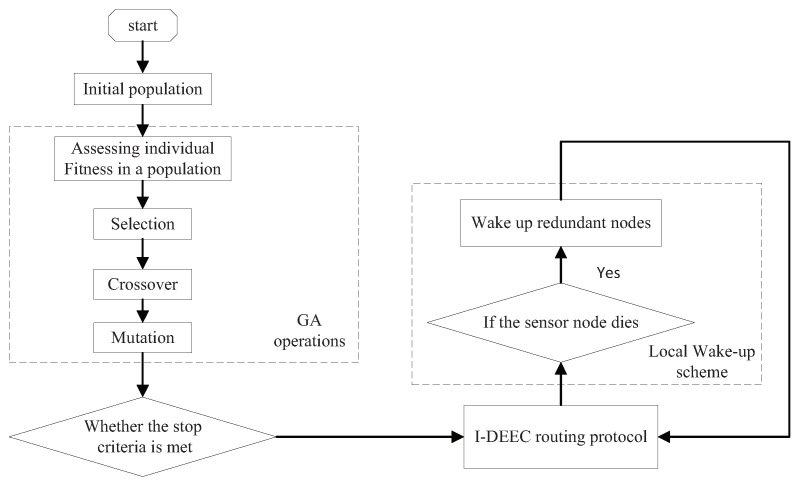
Flow chart of the combination of two algorithms.

In this study, we propose a solution that combines sensor node scheduling and routing optimization in order to improve the network lifetime. First of all, the minimum cover set is obtained using MCCA, which is also the basis of routing protocol design. Then the data is transmitted to the sink with minimum energy through the I-DEEC. When the sensor node dies, the wake-up scheme activates the relevant sleep sensor nodes to ensure network coverage and then starts a new round of data transmission. Figure 5 shows the flow chart.

## 6. Experimental Simulation

In this section, the performance of our proposed algorithms is verified. The results show that our algorithms are very promising.

### 6.1. MCCA Algorithm Simulation Results

#### 6.1.1. Parameter Setting

400 sensor nodes and 64 MTPs are randomly deployed in the area of 100 m × 100 m. To form a connected communication network, the sensing radius $R_{s}$ and the communication radius $R_{c}$ are set as 8.8375 m and 17.6750 m, respectively. The energy consumption rate of each activated sensor node is 0.1 J/s. While the sleeping node does not consume energy. The population size is assumed as 100, while the maximum genetic algebra is 15. Set the crossover rate as 0.7. The value of three weighting coefficients $\lambda_{1}$, $\lambda_{2}$, and $\lambda_{3}$ are 0.6, 0.2, 0.2, respectively.

#### 6.1.2. Results and Analysis

**Figure 6:**
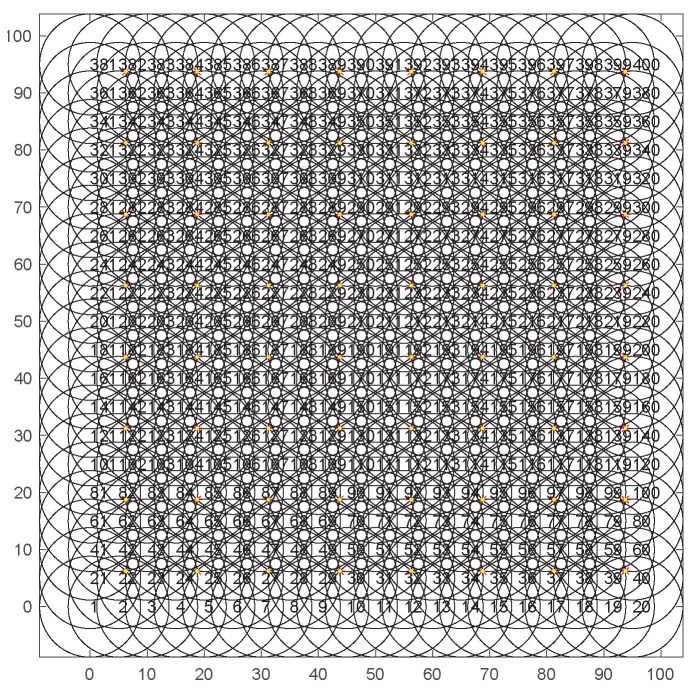
Distribution of 400 nuiform nodes and 64 monitoring target points.

**Figure 7:**
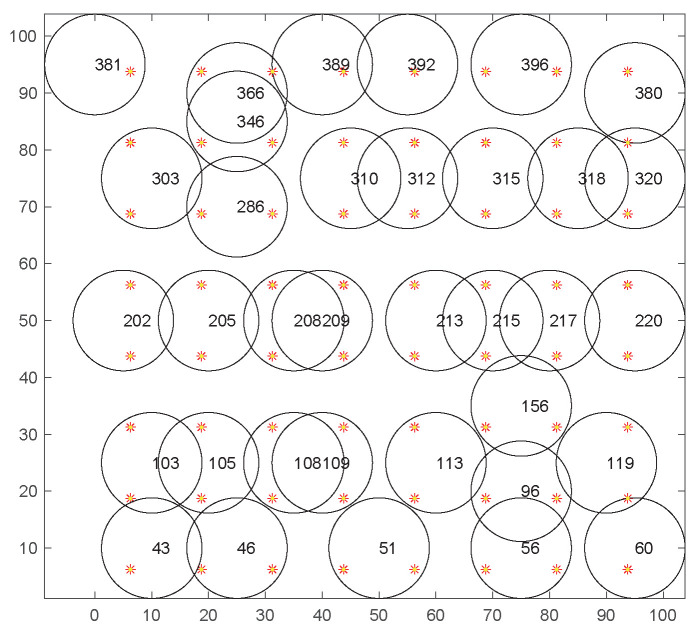
The coverage diagram after the 15th iteration.

Figure 6 shows a simulation scenario of uniform deployment, with a total of 400 sensor nodes and 64 MTPs set up. The black circles represent the sensing ranges of the sensor nodes, and the red stars represent MTPs. Figure 7 shows the coverage of the sensor nodes after 15 iterations. Only 35 sensor nodes need to be activated to cover all MTPs, in order to avoid the problem of redundant coverage.

**Figure 8:**
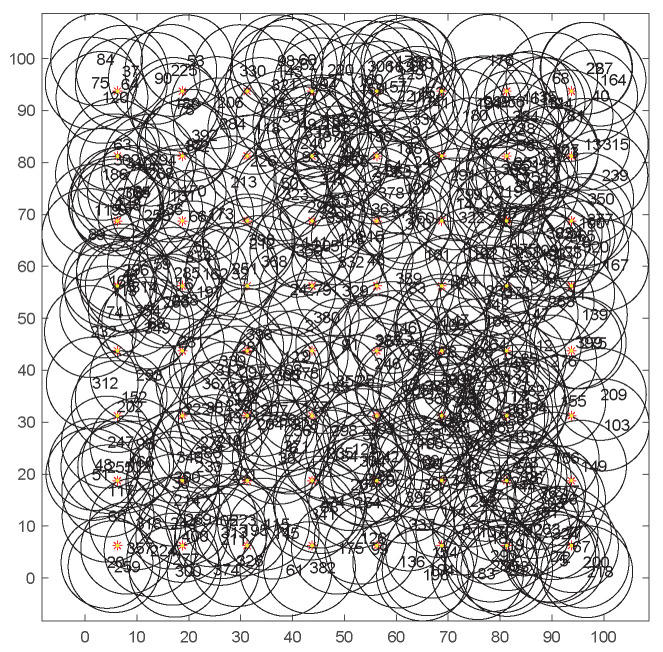
Distribution of 400 random nodes and 64 monitoring target points.

**Figure 9:**
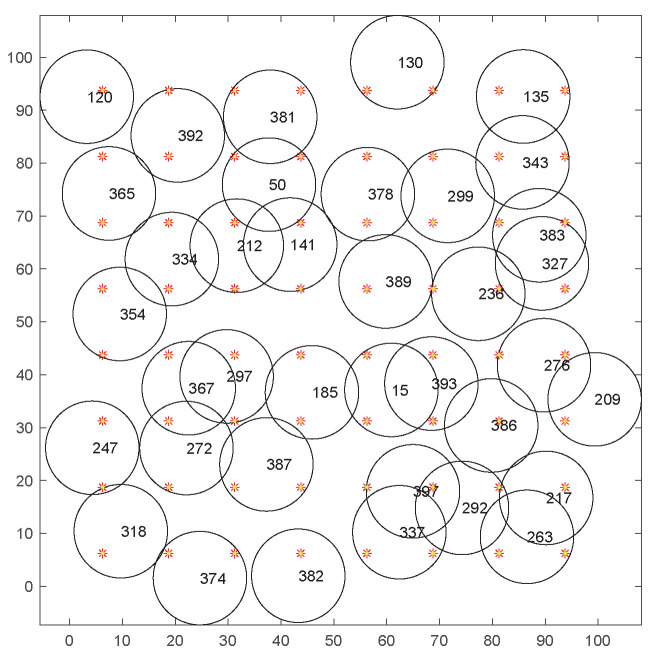
The coverage diagram after the 15th iteration.

Figure 8 shows the distribution of 400 random nodes and 64 MTPs. The coverage area of adjacent nodes overlaps with each other, which leads to the redundant coverage and energy waste. To extend the network life, the node scheduling should be optimized under the premise of ensuring coverage quality. Figure 9 shows the coverage condition of sensor nodes after 15 iterations by our algorithm. Only 37 sensor nodes are needed to be activated to cover all MTPs and the problem of the redundant coverage can be avoided.

Next, in order to evaluate the performance of the MCCA algorithm for reducing redundant sensor nodes, we compared it with I-GA (improved genetic algorithm) [10] and traditional GA.The same parameters are used for all algorithms.

**Figure 10:**
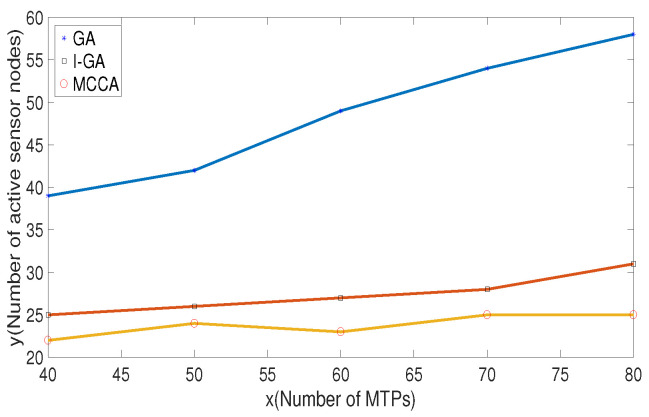
Comparison of the number of activated sensor nodes for different numbers of monitored target points (MTPs).

Figure 10 shows the number of sensor nodes that need to be activated for different numbers of MTPs. As the number of MTPs increases, the number of activated sensor nodes also increases. However, the number of sensor nodes selected to work in the MCCA algorithm proposed in this paper is lower than that of I-GA and traditional GA. It can be seen from the figure that, in order to cover 60 target points, the MCCA algorithm selects 23 sensor nodes, while I-GA and traditional GA require 27 and 47, respectively.

### 6.2. I-DEEC Simulation Results

The DEEC and IEE-LEACH [14] algorithm are used to compare with our proposed algorithm. To make the simulation more fair, the object used here is the number of dead nodes rather than the network life cycle. Table 1 shows the parameters of the experiment.

The impacts of different factors on the performance of mentioned algorithms are studied, which contains the number of dead nodes and throughput.

#### 6.2.1. Comparison of Changes in the Number of Dead Nodes with Time

**Figure 11:**
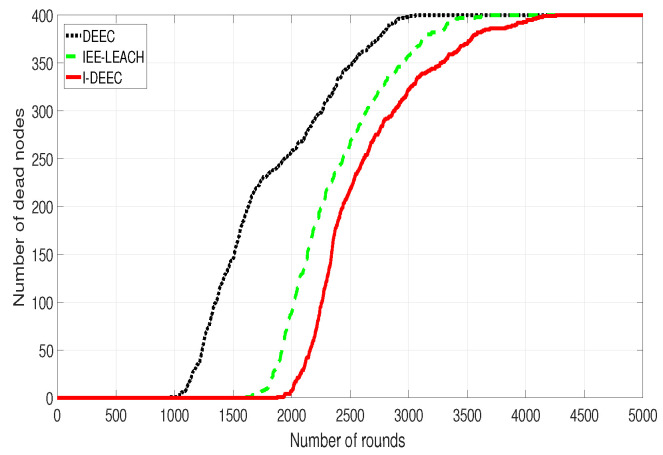
Comparison of changes in the number of dead nodes with time.

Figure 11 shows that, in DEEC and IEE-LEACH, the first dead node appears in the first 1000 rounds and 1600 rounds, respectively. However, in the proposed I-DEEC, this condition takes place in the 1800 rounds. It also can be noticed that, when all sensor nodes die, the rounds of the network operation by the DEEC and IEE-LEACH are 3000 and 3600, while that by our algorithm is 4300. The reason behind this phenomenon is that our algorithm takes the residual energy and the distance to the sink into consideration. When compared with two mentioned protocols, I-DEEC increases the network lifetime by about 19% to 43%. Hence, the performance of I-DEEC on the network is better than others.

#### 6.2.2. Comparison of Data Transmission

**Figure 12:**
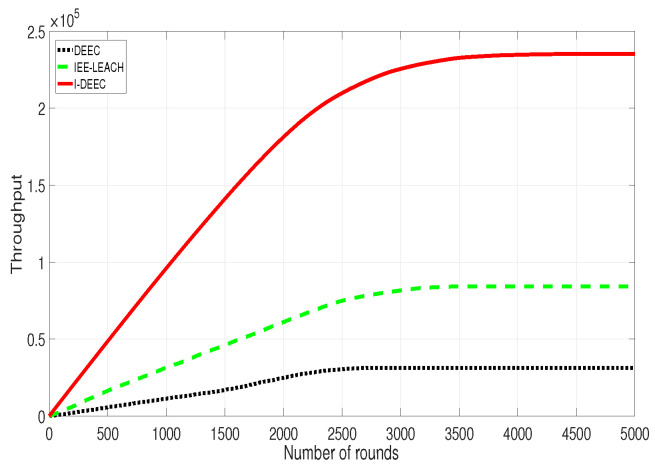
Comparison of data transmission.

In Figure 12, it can be found that the throughput by I-DEEC is much higher than the other two protocols. That is because I-DEEC has much higher energy efficiency than others. Therefore, with the limited energy, the network by our algorithm can send more data. At the same time, because the DEEC just considers the residual energy, the throughput by DEEC is minimum. For example, when the round is 2500, the throughput by DEEC, IEE-LEACH, and I-DEEC is , $0.3\times 10^{5}$, $0.8\times 10^{5}$ and $2.2\times 10^{5}$, respectively.

#### 6.2.3. Comparison of Network Energy Consumption

**Figure 13:**
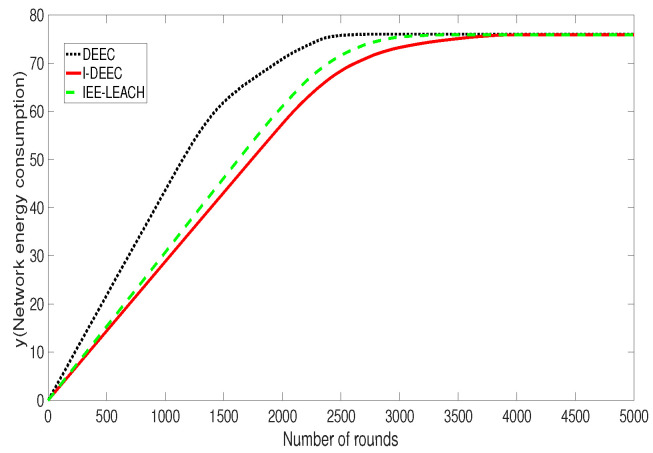
Comparison of network energy consumption.

Figure 13 shows the relationship between the total energy consumption of the three routing protocols and number of rounds. It can be seen that the total energy consumption of the I-DEEC protocol is less than the energy consumption of other protocols in the same round. Because the I-DEEC protocol adjusts the probability of a node becoming a cluster head, and designs a reasonable cluster size and the communication mode of sensor nodes around the sink, the proposed I-DEEC protocol further extends the life of the network.

### 6.3. The Lifetime of the Network

**Figure 14:**
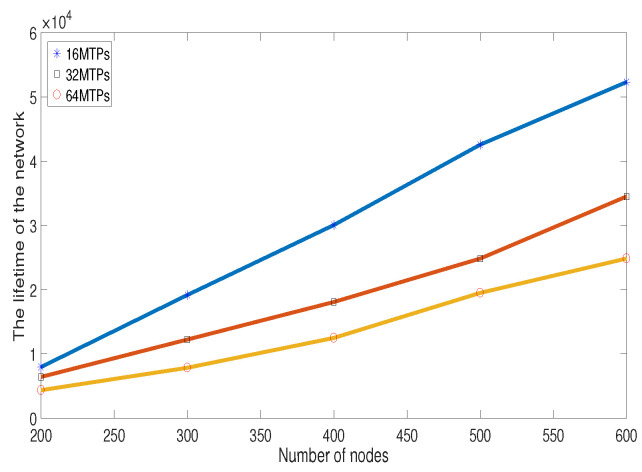
The lifetime of the network.

In Figure 14, the impacts of the number of sensor nodes and MTPs on the lifetime of the network are studied. The number of sensor nodes ranges from 200 to 600, while that of MTPs is set as 16, 32, and 64, respectively. The result shows that, when the number of MTPs is fixed, with the increase of the number of sensor nodes, the network lifetime will be increased. The reason is that the number of the redundant sensor nodes will also become larger. When there exists some dead node, more sensor nodes can be activated in order to keep the monitoring work continuous. At the same, it also can be found that the larger the number of MTPs is, the shorter the network lifetime is. This is because with the increase of the number of MTPs, the energy consumed on sensing will become higher, which will reduce the network lifetime.

## 7. Conclusions

This paper discusses the problem of maximizing the lifetime of the WSN. Through studying the characteristic of the energy consumption in WSNs, an efficient solution combining sensor node scheduling with routing protocol optimization is proposed. Firstly, in order to avoid the redundant coverage, the MCCA algorithm is proposed to find the minimum number of sensor nodes to monitor all MTPs. To keep monitoring MTPs continuously, a wake-up scheme is proposed in order to activate appropriate redundant node to replace the dead node. Based on the minimum coverage set, to optimize the energy consumed on the data communication, I-DEEC routing protocol is also proposed from three aspects. In CHs selection, by considering the energy and distance to the sink, the probability of nodes becoming CHs is adjusted. In cluster construction, some uneven clusters are also constructed to balance the energy consumption. Meanwhile, through comparing distances between the sensor node and the sink, and the CH, sensor nodes around the sink choose the communication way flexibly. The simulation results show that the proposed algorithms can improve the energy efficiency and the network lifetime, which is meaningful for the research on self-sustainable of the WSN. In this paper, we consider the ideal channel model. In the practical scenario, the current algorithm should make some changes to adapt to the unreliability of the wireless channel which will lead to transmission failure and sensing data missing. To guarantee the transmission of the sensing data, the proposed algorithm should combine with a certain efficient channel coding or retransmission scheme when it is applied to a real unreliable network.

## Figures and Tables

**Table 1:** Table of simulation experiment parameters.

Parameter	Numerical Value
$E_{0}$	0.5 J
$E_{fs}$	10 pJ/(bit · m^2^)
$E_{mp}$	0.0013 pJ/(bit · m^4^)
$E_{DA}$	5 nJ/bit
Weighting coefficient	α=0.5,β=0.5
